# New impact of the Journal of Applied Oral Science

**DOI:** 10.1590/1678-77572017ed002

**Published:** 2017

**Authors:** Karin Hermana Neppelenbroek, Vanessa Soares Lara

On June 14^th^, 2017, the Bauru School of Dentistry (FOB), University of São Paulo (USP) received with huge enthusiasm the great news of the 1.342 impact factor for the Journal of Applied Oral Science (JAOS) in the Journal Citation Reports^®^ (JCR), 2016 Science Edition. Out of the 90 journals indexed in this database in the Dentistry, Oral Surgery & Medicine category, JAOS is ranked at the 56^th^ place and the 3^rd^ quartile. Among all the Brazilian journals indexed in JCR, JAOS is the 11^th^ out of 111 journals and presents the greatest impact factor of dental journals.

JAOS' ascension trajectory is the result of hard work, initiated by former Editors-in-Chief and Associate Editors, with the collaboration of the technical team and unrestricted support of current and previous Deans of FOB-USP since its foundation. In 2003, the Revista da Faculdade de Odontologia de Bauru, under the scientific coordination of Dr. José Mauro Granjeiro, was renamed as the JAOS, with publication of English-language manuscripts only. Then, the editorial board was reformulated in order to broaden the geographic distribution of the *ad hoc* reviewers; important changes were made in the peer review guidelines; and set of recommendations accepted worldwide for manuscript presentation and reference format were adopted by the JAOS (“Uniform Requirements for Manuscripts Submitted to Journals”). Moreover, JAOS was indexed in the SciELO (Scientific Electronic Library Online), an electronic library covering a selected collection of Brazilian scientific journals, which allows free access to full manuscripts. Dr. Ricardo Marins de Carvalho, the Editor-in-Chief of the JAOS between 2005 and 2006, substantially increased the participation of foreign reviewers in the journal.

Part of the tremendous success that JAOS achieves today is attributed to the serious work begun with Dr. Carlos Ferreira Santos, who took over as the Editor-in-Chief of the JAOS from 2006 to 2014, focusing his administration mainly on journal indexation in international electronic databases. Between 2006 and 2007, JAOS was indexed in the following databases: SCOPUS, the world's largest abstract and citation multidisciplinary database of peer-reviewed literature and web sources, comprehending the fields of Life Sciences, Health Sciences (including all publications indexed in Medline/PubMed electronic database), Physical and Social Sciences; Cochrane Library, an electronic database consisting of updated medical information sources based on evidence and systematic reviews; and Science Citation Index Expanded (SCIE), published by Thomson Scientific, which qualified JAOS as an international publication since very few dental journals are indexed in this database, thus demonstrating its highly selective inclusion criteria and procedures for acceptance in the collection, available through Web of Science^®^ and the online version Sci Search^®^. As a result, JAOS was able to achieve a first impact factor in JCR in 2009 (0.386). Dr. Santos also introduced several changes in the journal's editorial management, mainly with the implementation of an online submission and editorial system in 2007, which increased the participation of foreign peer-reviewers and contributors. These changes as well the increased number of Associate Editors in 2010 automatically speeded up the review process and improved the impact factor to 0.797 in 2012. Dr. Gustavo Pompermaier Garlet was appointed as Co-Editor-in-Chief in 2013, and made an important contribution to the increase in the quality of manuscripts published in JAOS as well the improvement of the journal's peer-review system, which resulted in an impact factor of. 0.923. This success was subsequently maintained by Dr. Garlet and Dr. Karin Hermana Neppelenbroek, appointed as Editor-in-Chief and Co-Editor-in-Chief, respectively, between 2014 and 2016. Their administration focused on providing a fast turnaround time for manuscripts, and because of many changes and improvements that have been made and with the support of the whole editorial team, the average turnaround time for authors from manuscript submission to first decision was drastically reduced. The lower turnaround time for authors significantly contributed for the impact factor to have jumped to 1.117 in 2015.

Since last year, Dr. Neppelenbroek and Dr. Vanessa Soares Lara serve JAOS as Editor-in-Chief and Co-Editor-in-Chief, respectively, and a new panel of Associate Editors was formed, which includes colleagues from foreign institutions in order to meet recent criteria established by SciELO. Our management continues with the goal of further reducing the average response time to authors by providing detailed and quality reviews. We also keep the mission of increasing the international visibility of the JAOS.

We attribute the achievements of JAOS not only to the editorial board, but also to the important role of the authors for the submission of quality papers with high citation potential and the reviewers for performing a voluntary review work. We cannot forget to mention the unconditional support that JAOS receives from the Deans of FOB-USP, currently headed by Dr. Maria Aparecida de Andrade Moreira Machado and Vice-Dean Dr. Carlos Ferreira Santos as well the commitment and professionalism of our employees Valéria Cristina Trindade Ferraz, José Roberto Plácido Amadei, Deborah Schmidt Capella Junqueira, Sônia Cláudia Antonelli Pirola, Neimar Vitor Pavarini, Rubens Kazuo Kato, Camila Medina, Allan Rodrigo Dias and Zelma Batista Borges. Lastly, another major reason for JAOS’s success is the financial support provided over the last years by the Rectory of USP and the National Council for Scientific and Technological Development (CNPq).

The progressive increase of the impact factor produced a positive response, resulting in the submission of manuscripts that meet the standards of quality as well as in the establishment of more selective criteria and assessment strategies, which apply both for authors and for reviewers. Finally, this approach provided the required support and balance for the analysis of papers in the different areas of dental research. We look forward to continuing to receive excellent publications and high-quality reviews in fast turnaround time so that we have even more success rates in JAOS.

Yours sincerely,

Karin Hermana Neppelenbroek Editor-in-Chief, Journal of Applied Oral Science Vanessa Soares Lara Co-Editor-in-Chief, Journal of Applied Oral Science

